# Biofilm formation and antimicrobial resistance of *Pseudomonas aeruginosa* in cheese production systems

**DOI:** 10.1038/s41598-026-57335-2

**Published:** 2026-06-22

**Authors:** Noura K. Ahmed, Dalia Hamza, Shimaa S. Awaad, Ayah B. Abdel-Salam, Said S. Sallam

**Affiliations:** 1 Department of Food Hygiene and Control, Faculty of Veterinary Medicine, Cairo University, Giza Square, P.O Box 12211, Giza, Egypt; 2 Department of Zoonoses, Faculty of Veterinary Medicine, Cairo University, Giza Square, P.O Box 12211, Giza, Egypt; 3 Department of Veterinary Public Health, Faculty of Veterinary Medicine, Egyptian Chinese University, Cairo, Egypt

**Keywords:** Cheese, *Pseudomonas aeruginosa*, Multidrug resistance, Multiple antibiotic resistance, Biofilm, Food safety, Dairy contamination, Microbiology, Molecular biology

## Abstract

The emergence of drug-resistant *Pseudomonas aeruginosa* is an increasing global concern affecting human and animal health, food production systems, and environmental safety. This study investigated its occurrence, antimicrobial resistance, and biofilm-forming ability using phenotypic assays, and identify the biofilm-associated genetic markers in 120 cheese samples including 30 samples each of (Kariesh, Tallaga, Processed, and Romy) collected from various retail sources in Cairo and Giza, Egypt. *Pseudomonas aeruginosa* isolates were identified using both biochemical tests and molecular confirmation using 16 S rRNA gene. Antimicrobial susceptibility was evaluated using the disk diffusion method. Biofilm formation was assessed phenotypically through the microtiter plate and tube assays, while the biofilm-associated genes *pelA* and *pslA* were detected using PCR. *Pseudomonas aeruginosa* was detected in 18 samples (15%), and identification was confirmed through biochemical and molecular methods. Antimicrobial susceptibility testing revealed that 55% of isolates were extensively drug-resistant, while 45% exhibited multidrug resistance, with MAR indices ≥ 0.2 and an average MAR index of 0.68, indicating exposure to high-risk environments with frequent antibiotic use. Phenotypic assays showed strong biofilm-forming capabilities among isolates, with 100% positive by microtiter plate and 95% by tube method. Molecular screening further confirmed the prevalence of biofilm-associated genes, detecting *pelA* in 72% and *pslA* in 61% of isolates. Overall, *Pseudomonas aeruginosa* isolated from cheese samples exhibited substantial antimicrobial resistance and robust biofilm-forming ability, posing significant concerns to cheese quality and consumer health. These findings highlight the urgent need for continuous surveillance, improved dairy hygiene, effective sanitation, responsible antimicrobial practices, and alternative control strategies within the dairy sector to reduce potential public health hazards.

**Supplementary Information:**

The online version contains supplementary material available at 10.1038/s41598-026-57335-2.

## Introduction

Food spoilage represents a major challenge for the dairy industry, particularly in developing countries where inadequate processing, poor hygiene, and limited refrigeration are common. Microbial contamination leads to sensory deterioration, reduced product quality, reputational damage, and substantial economic losses for dairy producers^1^.

*Pseudomonas* spp. are major contributors to dairy spoilage due to their psychrotrophic nature and ability to grow at refrigeration temperatures. Although pasteurization effectively eliminates these bacteria, post-pasteurization contamination remains a concern because of environmental sources and inadequate sanitation practices^2^.

*Pseudomonas aeruginosa* is of particular concern in the dairy industry due to its metabolic versatility and ability to grow at refrigeration temperatures, facilitating its persistence along the food chain^3,4^. It is a major spoilage organism because of its production of thermoresistant lipolytic and proteolytic enzymes that degrade κ-, α-, and β-caseins, leading to sensory defects, reduced yield, and shortened shelf life of dairy products. Beyond spoilage, its strong biofilm-forming ability and antimicrobial resistance raise concerns regarding food quality, consumer safety, and public health, particularly because of the potential transmission of resistant strains through the food chain^5,6^.

*Pseudomonas aeruginosa* is a highly adaptable opportunistic pathogen and a prominent Gram-negative ESKAPE bacterium that poses a major clinical and public health challenge due to its extensive intrinsic, acquired, and adaptive resistance mechanisms^4,5^. These mechanisms facilitate persistence, dissemination, therapeutic failure, and rapid acquisition of resistance to multiple antimicrobial classes through antibiotic inactivation, target modification, reduced drug accumulation, chromosomal mutations, horizontal gene transfer, and biofilm formation^7–10^.

Antimicrobial resistance (AMR) has emerged as a critical global concern affecting human and animal health, food production systems, and environmental safety^11^. The widespread use of antibiotics in animal production has further accelerated the emergence and dissemination of resistant *P. aeruginosa* strains, increasing risks to both animal and public health^12^. High-risk multidrug-resistant (MDR) strains are now globally distributed and continue to pose substantial treatment and infection-control challenges^9,10^.

Multidrug-resistant *P. aeruginosa* is associated with significant morbidity, mortality, and healthcare costs worldwide. In the United States, the Centers for Disease Control and Prevention (CDC) reports more than 2.8 million antimicrobial-resistant infections and over 35,000 associated deaths annually^13^. Over the past decade, the CDC has consistently classified MDR *P. aeruginosa* as a serious public health threat, estimating approximately 32,600 infections, 2,700 associated deaths, and nearly US$767 million in annual healthcare costs^5,14,15^. Some MDR strains have acquired resistance to nearly all currently available antibiotics, severely limiting therapeutic options and complicating infection control efforts^13^.

The remarkable ability of MDR *P. aeruginosa* to survive under hostile environmental conditions further enhances its outbreak potential, particularly in healthcare settings where treatment failure and increased morbidity are common^10^. In epidemiological studies, the multiple antibiotic resistance (MAR) index is widely used as a rapid indicator of prior antimicrobial exposure and selective pressure within bacterial populations^16^.

In addition to antimicrobial resistance, biofilm formation is a key virulence trait of *Pseudomonas aeruginosa*. Biofilms are structured microbial communities embedded in an extracellular polymeric substance (EPS) matrix that promotes surface attachment and limits antibiotic penetration, thereby enhancing tolerance to antimicrobials and disinfectants^14^. Reduced metabolic activity within mature biofilms further diminishes antibiotic efficacy, contributing to persistent and recurrent infections^17,18^.

These biofilms act as persistent contamination reservoirs, promoting dairy spoilage and increasing food safety risks^19,20^. Clinically, biofilm formation is implicated in approximately 65% of healthcare-associated infections, highlighting its major public health relevance^21^.

Genetically, *P. aeruginosa* produces three principal exopolysaccharides—Pel, Psl, and alginate—that contribute to biofilm initiation, structural integrity, and protection against environmental stressors. The *pelA* gene encodes a cationic polysaccharide critical for biofilm establishment and maintenance, whereas *pslA* encodes a neutral exopolysaccharide involved in early surface attachment and intercellular aggregation^22–24^.

Biofilm formation markedly reduces the effectiveness of disinfectants commonly used in dairy farms, as the EPS matrix restricts disinfectant penetration and activity^25^. Disinfectants such as mercuric chloride, peracetic acid, and formaldehyde, although effective against planktonic cells, often show diminished efficacy against biofilm-embedded bacteria, necessitating higher concentrations and prolonged contact times to achieve comparable microbial inactivation^17,26^.

These findings highlight the growing challenge posed by multidrug-resistant *Pseudomonas aeruginosa* and underscore the need for coordinated global efforts, including strengthened surveillance, rational antibiotic use, and development of alternative antimicrobial strategies to limit the spread of resistance and protect public health in the post-antibiotic era^13^.

Therefore, the present study aims to investigate the prevalence of *Pseudomonas aeruginosa* in different cheese samples, with particular emphasis on the detection of multidrug-resistant strains and their biofilm-forming capabilities.

## Materials and methods

The study was conducted from September 2024 to July 2025 in two distinct geographic regions at Cairo and Giza Governorates in Egypt.

### Collection of samples

Initially, thirty dairy shops, supermarkets, and street vendors were selected for the collection of cheese samples located in Cairo and Giza Governorates, Egypt. A total of 120 cheese samples (500 g each) were randomly collected. The samples comprised 30 each of Kariesh, Tallaga, processed, and Romy cheeses. All samples were aseptically placed in sterile polyethylene bags, conveyed to the laboratory under refrigerated conditions (4 °C), and analyzed immediately upon arrival to minimize external contamination and microbial proliferation.

### Isolation and identification of *Pseudomonas aeruginosa*

Isolation and identification of *Pseudomonas aeruginosa* were performed as previously described^27,28^. Briefly, 25 g of each cheese sample was homogenized in 225 mL trisodium citrate solution (HiMedia, India), serially diluted, and plated onto Penicillin–Pimaricin Pseudomonas Agar (PP) (HiMedia M1788, India) supplemented with penicillin and pimaricin (100,000 IU/L; HiMedia FD264, India) and pimaricin (0.01 g/L; HiMedia FD265, India). Plates were incubated at 25 ± 1 °C for 48 ± 2 h. Presumptive colonies were identified based on typical pigmentation and grape-like odor. From each positive sample, five colonies were subjected to standard biochemical tests including oxidase and catalase activities, gelatin and polysaccharide hydrolysis, starch hydrolysis, pigment production on tryptic soy agar, carbohydrate fermentation profiles, and the ability to grow at 42 °C described by Al-Daghistani et al.^29^., and one biochemically confirmed *Pseudomonas aeruginosa* isolate was selected for further characterization.

## Molecular confirmation of *Pseudomonas aeruginosa* isolates

### Genomic DNA extraction

DNA was extracted from the selected biochemically confirmed isolates by inoculating them into Tryptone Soy Broth (TSB; HiMedia M1876, India) and incubating the cultures overnight at 30 °C. A 1.5-ml aliquot of each culture was transferred to Eppendorf tubes and centrifuged for 5 min, after which the supernatant was discarded, and the resulting cell pellet resuspended in 200 µl of TE buffer. The suspension was heated at 95 °C for 10 min to lyse the cells, cooled on ice, and centrifuged again for 5 min. The resulting supernatant served as the DNA template for PCR. DNA concentration and purity were evaluated using a NanoDrop spectrophotometer, and the extracted DNA was stored at − 20 °C until further molecular analysis^30,31^.

### PCR Identification

PCR amplification of the 16 S ribosomal ribonucleic acid gene was performed for molecular confirmation of *Pseudomonas* species and *Pseudomonas aeruginosa* using genus- and species-specific primers, following the protocol described by^22^. Molecular identification was validated using Pseudomonas aeruginosa (ATCC^®^ 27853™) as a positive control. Primer specificity was further confirmed in silico using the National Center for Biotechnology Information Primer-BLAST tool, which demonstrated high sequence similarity to the *Pseudomonas* species reference genome (accession number OR435040.1) and the *Pseudomonas aeruginosa* reference genome (accession number NC_002516.2).

The primer sequences used in this study and the expected sizes of their PCR amplicons are presented in Table 1.

**Table 1:** Primers sequences, target genes, amplicon sizes.

Bacteria	Gene	Primers sequences (5ʹ–3ʹ)	Amplified segment (bp)	Annealing temperature	Reference
*Pseudomonas* spp.	16 S rDNA	GACGGGTGAGTAATGCCTA CACTGGTGTTCCTTCCTATA	618 bp	54	^22,71^
*Pseudomonas aeruginosa*	16 S rDNA	GGGGGATCTTCGGACCTCA TCCTTAGAGTGCCCACCCG	956 bp	58	
Biofilm genes	*pelA*	CATACCTTCAGCCATCCGTTCTTC CGCATTCGCCGCACTCAG	786 bp	60	^22^
	*pslA*	TCCCTACCTCAGCAGCAAGC TGTTGTAGCCGTAGCGTTTCTG	656 bp	60	

Oligonucleotide and primer sequences specific for *Pseudomonas aeruginosa* investigated during the study.

### Antimicrobial susceptibility testing of *Pseudomonas aeruginosa*

Antimicrobial susceptibility of biochemically and molecularly confirmed *Pseudomonas aeruginosa* isolates was determined using the Kirby–Bauer disk diffusion method in accordance with CLSI guidelines^32^, and their reported use in human and veterinary practice within the region^33–35^, with *Pseudomonas aeruginosa* ATCC^®^ 27,853™ used as a quality control strain. Inhibition zones were measured and interpreted as susceptible (S), intermediate (I), or resistant (R). A total of nine antibiotics representing six antimicrobial classes were tested: aztreonam (30 µg), penicillin (10 µg), cefepime (30 µg), ceftazidime (30 µg), cefotaxime (30 µg), gentamicin (10 µg), ciprofloxacin (5 µg), levofloxacin (5 µg), and meropenem (10 µg).

Isolates that demonstrated resistance to at least one antimicrobial agent in three or more distinct antimicrobial classes were classified as multidrug-resistant (MDR) strains^36^.

Isolates that were resistant to all tested antibiotics except one or two antimicrobial agents across all evaluated classes (i.e., susceptible to no more than two antimicrobial groups) were classified as XDR (Extensively Drug-Resistant)^5^.

For each isolate, the MAR index was computed number of antibiotics to which the isolate is resistant / total number of antibiotics tested. MAR index values ≥ 0.2 were considered indicative of isolates originating from environments with frequent or excessive antibiotic use^37,38^.

### Screening of biofilm formation capacity

#### Phenotypic detection of biofilm formation

##### Tube method (TM)

Biofilm formation was evaluated qualitatively using the tube method described by^39^. Briefly, the 18 biochemically and molecularly confirmed *Pseudomonas aeruginosa* isolates were inoculated into polystyrene tubes containing tryptic soy broth (TSB; HiMedia M1876, India) supplemented with 1% glucose and adjusted to a 0.5 McFarland standard. Tubes were incubated overnight at 37 °C, after which the broth was discarded, and the tubes were washed three times with PBS (pH 7.2). The inner surfaces were then stained with 0.1% crystal violet for 15 min, rinsed again with PBS, and air-dried in an inverted position. Tubes containing only TSB with 1% glucose served as the negative control. Biofilm formation was deemed positive when a visible film adhered to the wall and bottom of the tube, and the intensity of staining was scored as weak (+), moderate (++), or strong (+++). All assays were performed in triplicate and repeated three times.

##### Microtiter plate assay (MTP)

Biofilm formation was quantified using the microtiter plate method described by^40^. Overnight *Pseudomonas aeruginosa* cultures grown in TSB at 37 °C were adjusted to a 0.5 McFarland standard, after which 20 µL of each culture was inoculated into 180 µL of fresh TSB in 96-well polystyrene microplates and incubated statically at 37 °C for 24 h. After incubation, wells were gently washed three times with 250 µL PBS to remove non-adherent cells, fixed with 200 µL methanol for 15 min, air-dried, and stained with 200 µL of 0.1% crystal violet. Following rinsing and drying, the bound dye was solubilized with 200 µL of 30% acetic acid, and absorbance was measured at 570 nm using an enzyme-linked immunosorbent assay (ELISA) microplate reader (BioTek, Model ELx800, Model ELx800, Srl, ItalyWinooski, Vermont, USA). Uninoculated TSB served as the negative control. For each isolate, the mean optical density (ODi) from three replicates was compared with the cut-off OD (ODc), defined as the mean negative control OD. Isolates were classified as non-adherent (ODi ≤ ODc), weakly adherent (ODc < ODi ≤ 2 × ODc), moderately adherent (2 × ODc < ODi ≤ 4 × ODc), or strongly adherent (ODi > 4 × ODc). The positive reference strain for biofilm formation was *Pseudomonas aeruginosa* ATCC^®^ 27,853™.

#### Molecular detection of biofilm-associated genes

PCR assays were performed to identify biofilm-associated genes (*pelA* and *pslA*) in all biochemical confirmed *Pseudomonas aeruginosa* isolates using previously extracted genomic DNA, following the protocol described by^22^. PCR reactions were carried out in a final volume of 25 µL, comprising 3 µL of template DNA, 12.5 µL of EmeraldAmp MAX PCR Master Mix (Takara, Japan), 0.5 µL of each primer (10 pmol/µL; Metabion, Germany), and PCR-grade water.

Thermal cycling was performed under the following conditions: an initial denaturation at 95 °C for 5 min, followed by 35 cycles of denaturation at 95 °C for 1 min, annealing at 60 °C for 1 min, and extension at 72 °C for 1 min, with a final extension at 72 °C for 5 min.

Each PCR run included a positive control and a negative control (nuclease-free water). The amplified products were resolved by electrophoresis on 1.5% agarose gels (Vivantis, Malaysia), stained, and visualized using a UV transilluminator. A 100-bp DNA ladder (Jena Bioscience GmbH, Germany) served as a molecular size marker for estimating amplicon lengths.

The primer sequences used in this study and the expected sizes of their PCR amplicons are presented in Table 1.

### Statistical analysis

Categorical variables were expressed as percentages with 95% confidence intervals and analyzed using Chi-square and Fisher’s exact tests as appropriate. Non-normally distributed continuous variables were compared using the Kruskal–Wallis test, correlations were assessed using Spearman’s rank coefficient, and agreement between biofilm measures and gene presence was evaluated using Cohen’s kappa. A p-value < 0.05 was considered statistically significant. All statistical analyses were performed using SPSS version 27.0 (IBM Corp., Armonk, NY, USA).

## Results

### Occurrence of *Pseudomonas aeruginosa* in examined cheese samples

Out of the 120 examined cheese samples, *Pseudomonas* spp. were isolated from 90 samples (75%). Biochemical identification of the presumptive isolates revealed that 18 samples (15%) were positive for *Pseudomonas aeruginosa*, 24 samples (20%) for *Pseudomonas fluorescens*, and 48 samples (40%) for *Pseudomonas putida*. The prevalence of *Pseudomonas aeruginosa* varied among cheese types, with an overall prevalence of 15% (95% CI: 9.6–22.8%). The highest occurrence was detected in Tallaga cheese (7/30; 23.3%), followed by processed cheese (5/30; 16.7%), Kariesh cheese (4/30; 13.3%), and Romy cheese (2/30; 6.7%). Presumptive *Pseudomonas aeruginosa* colonies exhibiting typical morphology on selective media were further confirmed by PCR targeting the 16 S rRNA gene. Accordingly, all eighteen isolates were definitively identified as *Pseudomonas aeruginosa* based on 16 S rDNA sequencing.

### Antimicrobial resistance patterns of *Pseudomonas aeruginosa* isolates

**Fig. 1:**
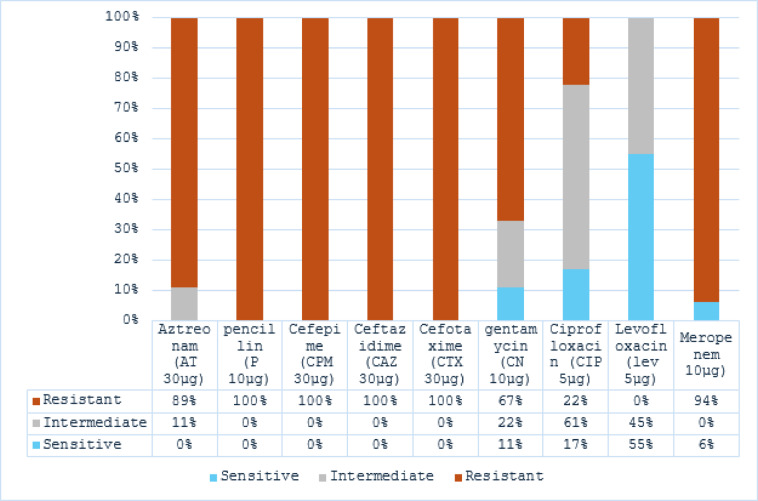
Phenotypic antimicrobial resistance patterns of *Pseudomonas aeruginosa* (*n* = 18) isolated from cheese samples. Resistance and sensitivity profiles of *Pseudomonas aeruginosa* isolates (*n* = 18) determined by Kirby-Bauer disc diffusion method using antibiotics from different classes to assess percentage of antimicrobial resistance patterns.

Antimicrobial susceptibility testing was conducted through the standard disc diffusion method revealed that all molecularly confirmed *Pseudomonas aeruginosa* isolates showed resistance to one or more of the tested antimicrobial agents. Notably, 100% of the isolates were resistant to penicillin (P, 10 µg), cefepime (CPM, 30 µg), ceftazidime (CAZ, 30 µg), and cefotaxime (CTX, 30 µg). In contrast, levofloxacin (LEV, 5 µg) demonstrated the highest antimicrobial activity, with 55% of isolates showing susceptibility. Lower susceptibility rates were observed for ciprofloxacin (CIP, 5 µg) and gentamicin (CN, 10 µg), with susceptibility percentages of 17% and 11%, respectively. Detailed susceptibility patterns for the remaining antimicrobials are presented in Fig. 1.

A total of 45% of the isolates were classified as MDR strains because they exhibited resistance to at least three classes of antibiotics. Since 55% showed no resistance to at least two classes of antibiotics, they were classified as extensively drug-resistant (XDR).

The distribution of antibiotic resistance profiles is summarized in Table 2. Of the 18 molecularly confirmed *Pseudomonas aeruginosa* isolates, 8 isolates (45%) were identified as multidrug-resistant (MDR), demonstrating resistance to at least one antimicrobial agent in three or more antimicrobial classes and 10 isolates (55%) were identified as extensively drug resistant (XDR), that resist all tested antimicrobial agents except one or two from all the classes evaluated. Furthermore, all isolates (100%) had a MAR index ≥ 0.2, with values ranging from 0.66 to 0.88 and a mean MAR index of 0.68, suggesting exposure to high-risk environments characterized by intensive or recurrent antibiotic use.

**Table 2:** Antibiotic resistance profiles, MAR Index, and MDR of *Pseudomonas aeruginosa* (*n* = 18) isolated from cheese samples.

Isolates	Cheese type	Antimicrobial resistance phenotype^®^	No. of resistant antibiotics	MAR Index	MDR
1	Processed	AT, P, CPM, CAZ, CTX, MRP	6	0.66	+
2	Kariesh	AT, P, CPM, CAZ, CTX, MRP	6	0.66	+
3	Kariesh	P, CPM, CAZ, CTX, MRP, CN	6	0.66	+
4	Tallaga	AT, P, CPM, CAZ, CTX, MRP	6	0.66	+
5	Tallaga	AT, P, CPM, CAZ, CTX, MRP	6	0.66	+
6	Tallaga	AT, P, CPM, CAZ, CTX, MRP	6	0.66	+
7	Processed	AT, P, CPM, CAZ, CTX, CN	6	0.66	+
8	Processed	AT, P, CPM, CAZ, CTX, MRP	6	0.66	+
9	Kariesh	AT, P, CPM, CAZ, CTX, MRP, CN	7	0.77	+
10	Tallaga	AT, P, CPM, CAZ, CTX, MRP, CN	7	0.77	+
11	Tallaga	AT, P, CPM, CAZ, CTX, MRP, CN	7	0.77	+
12	Tallaga	AT, P, CPM, CAZ, CTX, MRP, CN	7	0.77	+
13	Processed	AT, P, CPM, CAZ, CTX, MRP, CN	7	0.77	+
14	Romy	AT, P, CPM, CAZ, CTX, MRP, CN	7	0.77	+
15	Romy	AT, P, CPM, CAZ, CTX, MRP, CN	7	0.77	+
16	Kariesh	AT, P, CPM, CAZ, CTX, MRP, CIP, CN	8	0.88	+
17	Tallaga	AT, P, CPM, CAZ, CTX, MRP, CIP, CN	8	0.88	+
18	Processed	AT, P, CPM, CAZ, CTX, MRP, CIP, CN	8	0.88	+

*MAR index = No of antibiotics to which the isolate is resistant / total No of antibiotics tested.

MDR= Multidrug resistant.

### Phenotypic and genotypic biofilm profiles of *Pseudomonas aeruginosa* isolates

Biofilm-forming capacity of *Pseudomonas aeruginosa* isolates was assessed through two phenotypic assays: the microtiter plate (MTP) assay, considered the gold standard, and the tube method (TM).

**Fig. 2:**
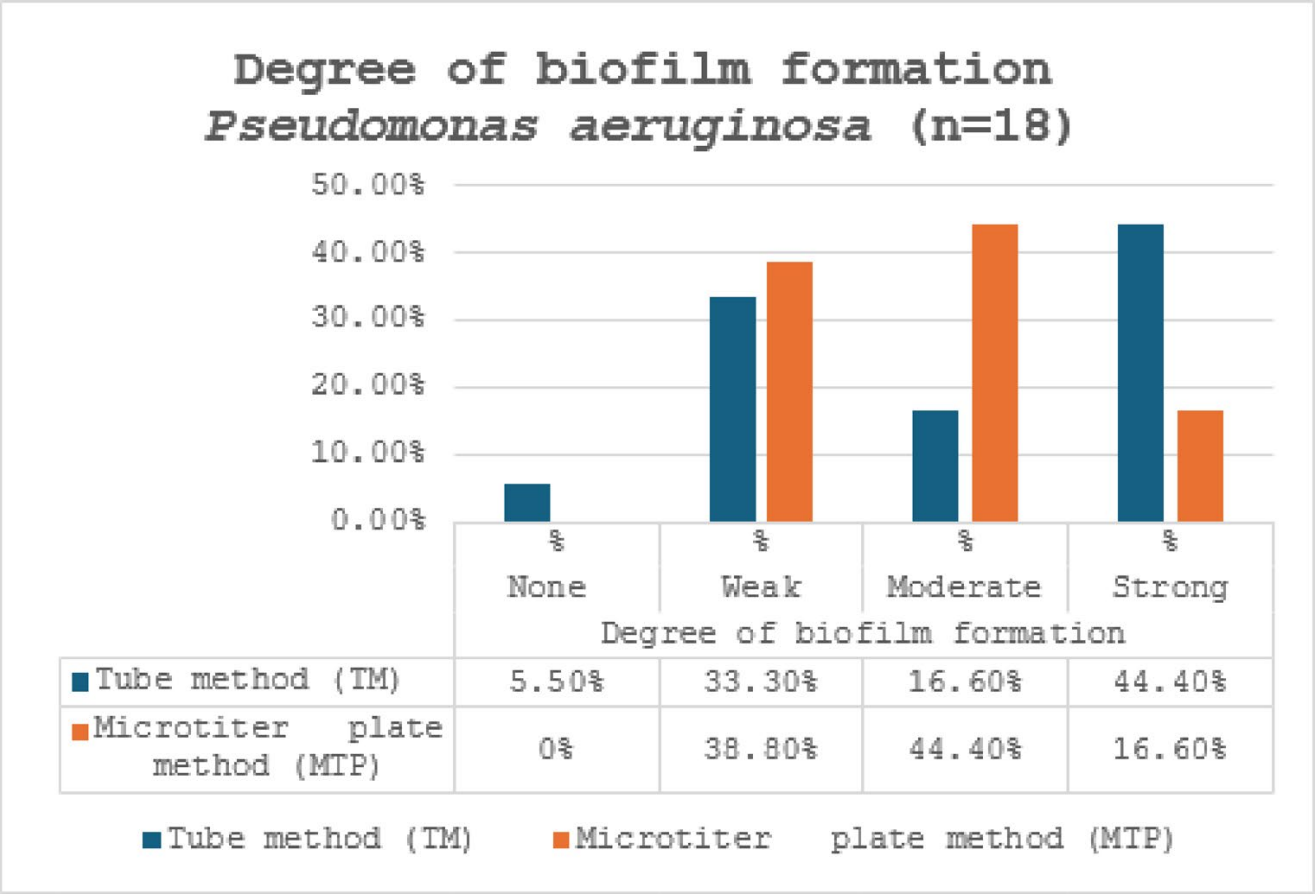
Phenotypic biofilm formation profiles of *Pseudomonas aeruginosa* isolates (*n* = 18). Assessment of biofilm formation by two methods, showing isolates categorized as non-biofilm producers and biofilm producers classified into strong, moderate, and weak degrees.

Using the MTP assay, all 18 isolates (100%) were identified as biofilm producers at varying levels. Specifically, 38.8% of the isolates were classified as weak biofilm producers, 44.4% demonstrated moderate biofilm producers, and 16.6% exhibited strong biofilm producers (Fig. 2).

In contrast, the tube method identified 94% of the isolates as biofilm producers. Among these, 33.3% were categorized as weak biofilm producers, 16.6% as moderate producers, and 44.4% as strong biofilm producers.

PCR analysis targeting the biofilm-associated genes (*pelA* and *pslA*) was conducted on isolates that were phenotypically confirmed as biofilm producers. The results revealed that 13 isolates (72%) tested positive for the *pelA* gene, while the remaining 5 isolates (28%) were negative. Regarding the *pslA* gene, 11 isolates (61%) tested positive, whereas 7 isolates (39%) were negative.

### Statistical analysis

A Chi-square test showed no significant association was observed between cheese type and the occurrence of *Pseudomonas aeruginosa* (χ² = 3.71, *p* > 0.05). Although strong biofilm production was more frequent among XDR than MDR isolates, no significant association was found between resistance category and biofilm strength (χ² = 0.257, *p* = 0.879), a result supported by Fisher’s exact test (*p* = 0.33). MAR index values did not differ significantly across cheese types (H = 0.97, *p* = 0.81) using the Kruskal–Wallis test. Spearman analysis showed a strong positive correlation between MAR index and the number of antibiotics resisted (*r* = 0.91, *p* < 0.01), and moderate positive correlations between biofilm formation and both MAR index (*r* = 0.52, *p* < 0.05) and antibiotic resistance count (*r* = 0.54, *p* < 0.05). Cohen’s kappa indicated poor agreement between biofilm assessment methods (κ = −0.23) and between phenotypic biofilm intensity and the presence of *pelA* (κ = 0.018, *p* = 0.814) or *pslA* (κ = 0.085, *p* = 0.280), suggesting that gene presence alone does not reliably predict biofilm-forming intensity.

### Cluster analysis of phenotypic biofilm formation, genotypic biofilm characteristics, and antimicrobial resistance profiles

**Fig. 3:**
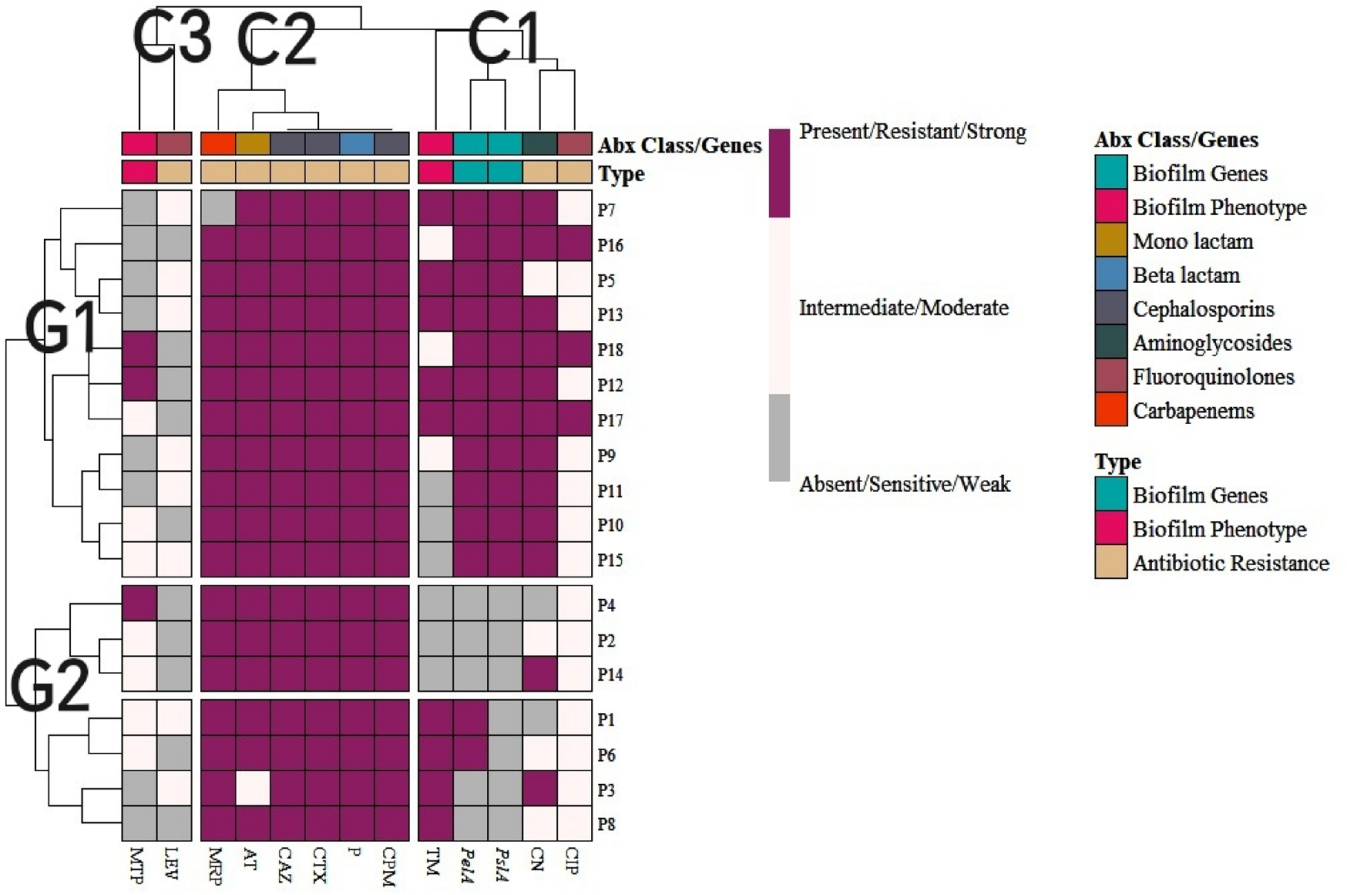
Heatmap of *Pseudomonas aeruginosa* strains clustered according to phenotypes of antimicrobial resistance (disc diffusion test) and both biofilm phenotypic methods and biofilm genes. Phenotype is represented as resistant/ strong (dark purple), intermediate/ moderate (pale pink), and sensitive/ weak (grey). Biofilm genotype is indicated as present (dark purple) and absent (grey). Each row represents an isolate, coded as P (*Pseudomonas aeruginosa*).

Hierarchical cluster analysis (Fig. 3) grouped the 18 *Pseudomonas aeruginosa* isolates into two main groups (G1 and G2) based on biofilm-associated gene presence, phenotypic biofilm profiles, and resistance to nine antimicrobial agents. The upper section of the heatmap (C1–C3) represents the distribution of biofilm genes, biofilm phenotypic patterns, and antimicrobial resistance patterns, respectively.

Isolates P.2 and P.14 clustered closely, sharing identical biofilm gene profiles (*pelA* and *pslA*) and similar resistance patterns, although P.14 was resistant to one additional antibiotic. The MDR isolates P.1 and P.6 showed identical genotypic and phenotypic characteristics, including biofilm gene carriage, biofilm phenotype, and resistance profile. Similarly, the XDR isolates P.12 and P.18 clustered together with identical biofilm gene profiles and extensive resistance patterns, with P.18 exhibiting resistance to one additional antibiotic. Isolate P.17 clustered closely with P.18, sharing the same biofilm gene profile and comparable antimicrobial resistance characteristics (Fig. 3).

**Table 3:** Biofilm phenotype and pathotype of *Pseudomonas aeruginosa* (*n* = 18) isolated from cheese samples.

Isolates	Biofilm phenotype^®^		Biofilm genes	
	MTP	TM	pelA	pslA
1	++	+++	+	-
2	++	+	-	-
3	+	+++	-	-
4	+++	+	-	-
5	+	+++	+	+
6	++	+++	+	-
7	+	+++	+	+
8	+	+++	-	-
9	+	++	+	+
10	++	+	+	+
11	+	+	+	+
12	+++	+++	+	+
13	+	+++	+	+
14	++	+	-	-
15	++	+	+	+
16	+	++	+	+
17	++	+++	+	+
18	+++	++	+	+

TM = Tube method.

MTP = Microtiter plate method.

## Discussion

Dairy products, particularly cheese, are highly susceptible to contamination by spoilage microorganisms such as *Pseudomonas aeruginosa*, which may enter milk and milk-based products as a result of inadequate production practices, processing failures, or unhygienic handling conditions^41^. The detection of *P. aeruginosa* in dairy products is widely regarded as a strong indicator of poor hygienic status, as it often reflects ineffective sanitation procedures, substandard water quality, or post-processing contamination within the dairy production environment^42^.

The present study revealed a notable occurrence of *Pseudomonas aeruginosa* across the examined cheese samples, likely influenced by storage-related factors such as temperature, duration, and environmental conditions that support bacterial survival and growth^43^. In addition, its high metabolic versatility, environmental adaptability, and ability to grow at refrigeration temperatures enhance its persistence in dairy environments^6,44^. Although no significant differences were observed among cheese types, the presence of *P. aeruginosa* in all varieties suggests that contamination is more closely related to hygiene, handling, or processing practices than to cheese-specific characteristics.

The higher prevalence observed in fresh cheeses such as Tallaga and Kariesh may reflect their higher moisture content, lower salt levels, and lack of prolonged ripening, which favor bacterial survival and growth. In contrast, the lower prevalence in Romy cheese is likely associated with its longer ripening period, reduced water activity, and higher salt content, creating less favorable conditions for microbial persistence^45,46^. Similar prevalence patterns have been reported by Atia et al. and Aziz et al.^6,25^. These findings further support the ability of *P. aeruginosa* to persist in dairy processing environments.

Additionally, the use of raw milk and refrigerated storage prior to heat treatment may enhance survival and dissemination along the dairy production chain. Lower prevalence rates were reported by Deiab et al., who analyzed bulk tank milk and dairy products from Menofia Governorate, Egypt, and by Salem et al., who examined mastitis milk samples from dairy farms in Dakahlia Governorate, Egypt^47,48^. In contrast, higher contamination levels were observed by Elgharabawy et al. in Kariesh cheese from El Beheira Governorate and by Gamal et al. in various dairy products collected from markets and farms in Dakahlia Governorate, Egypt^28,49^.

*Pseudomonas aeruginosa* and other spoilage or pathogenic bacteria may be transmitted to consumers through fresh dairy products, particularly when hygiene during processing and handling is inadequate. The risk is greater for ready-to-eat cheeses, especially those produced from raw milk, where low acidity and salt content favor bacterial survival. Variations in contamination likely reflect differences in hygiene and processing conditions across dairy environments. Molecular analysis based on 16 S rRNA gene detection identified all isolates as *P. aeruginosa*, consistent with previous reports^28,48,50^, although the limited discriminatory power of this marker within the *Pseudomonas* genus should be acknowledged.

All *Pseudomonas aeruginosa* isolates exhibited resistance to penicillin and cefotaxime, consistent with the organism’s intrinsic resistance and the limited activity of these agents^51^. The extensive use of β-lactam antibiotics has contributed to the emergence of β-lactam-resistant *P. aeruginosa*, increasing both clinical risk and economic burden^52^. Complete resistance to the antipseudomonal cephalosporins ceftazidime and cefepime was observed and is likely associated with AmpC β-lactamase hyperproduction and efflux pump upregulation. While basal AmpC expression is typically insufficient to confer resistance, the pattern detected here suggests inducible AmpC activity, also known as Pseudomonas-derived cephalosporinase (PDC)^53,54^.

In high-risk epidemic clones, mutation-driven and horizontally acquired β-lactam resistance mechanisms may coexist, leading to difficult-to-treat resistance (DTR) phenotypes^54^. The resistance profiles observed in this study are consistent with advanced mechanisms, including enhanced β-lactamase activity, efflux overexpression, and biofilm-associated tolerance, commonly linked to multidrug resistance and prolonged β-lactam exposure^55^. The lack of molecular characterization (e.g., detection of resistance genes or efflux regulators) represents a limitation, as genotype–phenotype integration is essential to elucidate underlying resistance determinants. These findings align with reports by Salem et al. on mastitis milk isolates in Egypt^48^. In contrast, lower resistance rates to ceftazidime, cefepime, and meropenem were reported by Gamal et al.^49^, likely reflecting differences in antimicrobial usage and selective pressure across study settings.

All isolates recovered in this study were classified as extensively drug-resistant (XDR) or multidrug-resistant (MDR). This pattern may reflect extensive antibiotic use in dairy farming and horizontal acquisition of resistance determinants, raising concerns about potential transmission to humans through contaminated cheese^12,56^. Higher MDR rates were reported by Salem et al.^48^, whereas lower rates were observed by Badawy et al.^57^, suggesting variability related to antibiotic stewardship and environmental exposure.

All *Pseudomonas aeruginosa* isolates exhibited MAR index values greater than 0.5, indicating origin from high-risk environments with frequent antibiotic use. Consistent with previous reports^48^, these findings support the utility of the MAR index as an indicator of antimicrobial pressure and suggest that dairy environments may serve as reservoirs for antibiotic-resistant bacteria^58^, despite lower MAR values reported in other studies by Gamal et al.^49^ and Ibrahim et al.^12^.

Phenotypic assays as shown in Table 3 showed that most *Pseudomonas aeruginosa* isolates were capable of biofilm formation at varying intensities, supporting their ability to adhere to surfaces and tolerate antimicrobial exposure. Lower proportions of biofilm-forming isolates were reported by Aziz et al. and Deiab et al.^25,47^, whereas comparable or higher prevalence was observed in other studies^48,59^. The association between biofilm-forming capacity and multidrug resistance underscores the clinical and environmental relevance of *P. aeruginosa*, as biofilms promote persistence under antimicrobial pressure.

Within biofilms, close cell-to-cell contact enhances horizontal gene transfer and promotes physiological states that reduce antimicrobial susceptibility^60^. Biofilm-associated resistance differs from planktonic resistance because the EPS matrix limits antibiotic penetration, while metabolic heterogeneity promotes tolerance through slow-growing and persister cells^61,62^. Biofilm growth is associated with altered gene expression, including efflux pump upregulation and stress response activation, coordinated by quorum sensing systems in *P. aeruginosa*. In dairy and food processing environments, biofilms on equipment surfaces act as persistent reservoirs of resistant bacteria, facilitating repeated contamination and linking biofilm formation to antimicrobial resistance and food safety concerns^61,63^.

Agreement analysis between the microtiter plate (MTP) and tube methods (TM) showed poor concordance, reflecting inherent methodological differences. The MTP assay provides a quantitative and more reproducible assessment of biofilm biomass, whereas the TM relies on subjective visual interpretation and is prone to variability. Similar weak agreement has been reported previously, indicating that these methods should not be used interchangeably for biofilm detection or grading^64,65^.

The relatively high detection rates of the biofilm-associated genes *pelA* and *pslA* highlight their importance in biofilm development in *P. aeruginosa *as shown in Table 3. The prevalence of *pelA* in this study was lower than that reported by Lutfi N. et al.^66^, who detected this gene in all *P. aeruginosa* isolates recovered from mastitic milk of lactating Dorper sheep in Malaysia and from dairy-associated and clinical sources in Egypt, but higher than that reported by El Shora et al.^67^, who investigated milk samples collected from different markets in Egypt. Similarly, *pslA* prevalence was lower than that reported by El Shora et al.^67^ yet exceeded the rate documented by Elshazely et al.^50^ in raw milk and dairy products obtained from retail outlets and markets in Egypt. Variations among studies likely reflect differences in sample sources, methodological approaches, and genetic diversity among *P. aeruginosa* populations.

The presence of phenotypic biofilm formation in isolates lacking *pelA* and *pslA* highlights the multifactorial regulation of biofilm development in *P. aeruginosa*. Biofilm formation is not solely dependent on the *pel* and *psl* operons, as alternative determinants such as *algD*-mediated alginate production and quorum sensing systems (las and rhl) can independently or synergistically drive biofilm initiation and maturation^68,69^. This functional redundancy explains the weak agreement between phenotypic biofilm intensity and *pelA* or *pslA* carriage and indicates that gene presence alone is an unreliable predictor of biofilm-forming capacity, which is shaped by regulatory complexity, gene expression dynamics, and environmental factors^61,70^.

## Conclusion

This study demonstrates the presence of *Pseudomonas aeruginosa* in different cheese types, which may reflect potential deficiencies in hygienic practices during milk handling, processing, and storage. The isolates exhibited notable antimicrobial resistance, including multidrug-resistant and extensively drug-resistant profiles, alongside strong biofilm-forming capacity, which could contribute to their persistence in dairy environments. These findings highlight dairy products may act as potential reservoirs of resistant *P. aeruginosa* and emphasize the importance of improved hygiene measures, prudent antimicrobial use, and continuous surveillance to ensure food safety within a One Health context.

## Supplementary Information

Below is the link to the electronic supplementary material.

Supplementary Material 1

## Data Availability

Datasets are available from the corresponding author upon reasonable request.
