# Supplementary material for: Biofilm formation and antimicrobial resistance of Pseudomonas aeruginosa in cheese production systems
Source: Sci Rep. 2026 Jun 22;16:19384. doi: 10.1038/s41598-026-57335-2 (PMC13287804; doi:10.1038/s41598-026-57335-2)

Representative agarose gel electrophoresis images showing PCR amplification of target genes in *Pseudomonas aeruginosa* isolates.
(A) Amplification of the 16S rRNA gene confirming molecular identification of *P. aeruginosa*. (956 bp).


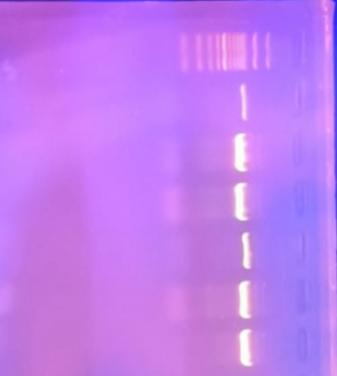

(B) PCR amplification of the biofilm‑associated *pelA* gene (786 bp).


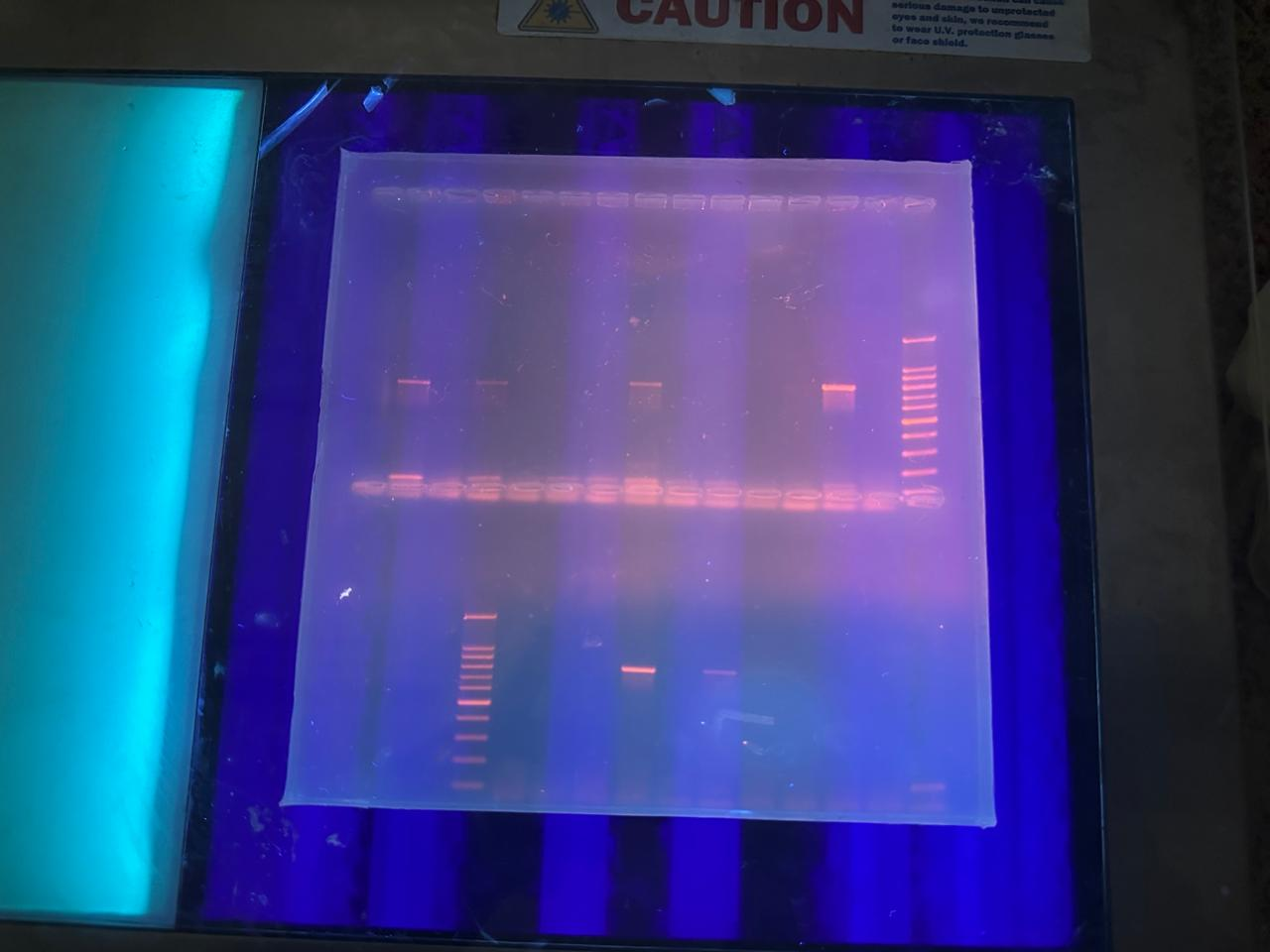

(C) PCR amplification of the biofilm‑associated *pslA* gene (656 bp).


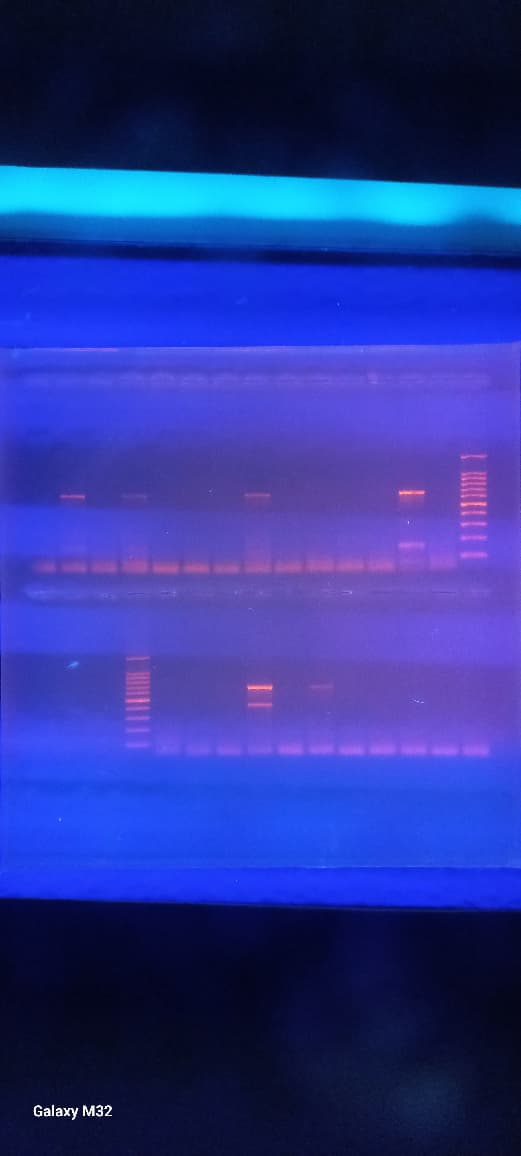

Supplement: Supplementary file 1 — Supplementary Material 1 [file 41598_2026_57335_MOESM1_ESM.docx]
